# Persistent Challenges: A Comprehensive Review of Persistent Postural-Perceptual Dizziness, Controversies, and Clinical Complexities

**DOI:** 10.7759/cureus.60911

**Published:** 2024-05-23

**Authors:** Jorge Madrigal, Andrés Felipe Herrón-Arango, Maria J Bedoya, Jairo Cordero Chen, Melissa Castillo-Bustamante

**Affiliations:** 1 Otoneurology, Centro de Vértigo y Mareo, Mexico City, MEX; 2 Otolaryngology, Universidad Pontificia Bolivariana, Medellín, COL; 3 Otolaryngology, Hospital Dr. Tony Facio Castro, Caja Costarricense de Seguro Social, Limón, CRI; 4 Medicine, Universidad Pontificia Bolivariana, Medellín, COL

**Keywords:** cognitive-behavioral therapy, vestibular rehabilitation, chronic subjective dizziness, persistent postural perceptual dizziness, vestibular disorders

## Abstract

Persistent postural-perceptual dizziness (PPPD) is a chronic and disabling disorder characterized by persistent dizziness, unsteadiness, and imbalance. It often arises without an identifiable cause and is exacerbated by upright posture, active or passive movement, and exposure to moving or complex visual stimuli. This complex pathophysiology and the psychological dimensions of its symptomatology pose a significant challenge to clinicians. PPPD presents diagnostic challenges and a lack of standardized treatment options, underscoring the need for multidisciplinary approaches encompassing pharmacotherapy, vestibular rehabilitation, and psychological interventions for effective management. Bridging the gaps in understanding PPPD requires collaborative efforts across disciplines, emphasizing integrated research approaches and patient support networks to enhance care and improve outcomes. This review explores the challenges, controversies, and clinical complexities of PPPD, highlighting the importance of a patient-centered approach.

## Introduction and background

Persistent postural-perceptual dizziness (PPPD) is a functional, multifaceted medical condition characterized by a prolonged, often daily, sense of dizziness, unsteadiness, or imbalance [1,2]. Most of the epidemiological data on PPPD are derived from studies on phobic postural vertigo, visual vertigo, and chronic subjective dizziness [1,2]. The estimated prevalence of PPPD among patients with vestibular symptoms is 15-20%, and the estimated incidence following acute or episodic vestibular disorders is about 25% of patients [3]. The average age of patients presenting for evaluation is in the mid-40s, and female patients are predominant [3]. Only a minority of patients experience spontaneous resolution of symptoms [3].

This condition goes beyond typical vertigo and is not exclusively triggered by specific head movements [1,2]. The exploration of PPPD involves an extensive review that delves into various facets, encompassing diagnostic criteria, clinical presentations, and underlying pathophysiological mechanisms [1,2]. Despite the existence of the Bárány Society criteria, a major factor adding to the complexity of PPPD is the lack of universally accepted diagnostic and treatment guidelines [3]. This uncertainty has sparked debates within the medical community [3]. Some doctors believe that stricter criteria are necessary to ensure accurate diagnosis, while others think a broader approach is better to capture the wide range of PPPD symptoms [3]. This ongoing discussion makes it difficult to create a standardized way to diagnose PPPD, which can be confusing for those new to the topic [3]. Moreover, understanding the pathophysiological basis of PPPD remains elusive, adding another layer of complexity [4]. The interplay between vestibular, proprioceptive, and visual sensory systems and potential central nervous system contributions raises intricate questions [4]. 

Managing PPPD involves various challenges, often requiring a holistic approach that includes pharmacotherapy, vestibular rehabilitation, and psychological interventions [4-6]. Additionally, integrating mental health aspects is essential, given the strong link between PPPD and conditions like anxiety and depression, which adds another layer of complexity to patient care [5,6]. This review aims to provide a comprehensive overview of PPPD, incorporating the most recent literature to offer insights into the current state of knowledge on the topic [3-6].

## Review

Understanding PPPD

PPPD is a multifaceted disorder characterized by persistent dizziness and a sensation of unsteadiness, often exacerbated by upright posture or movement [1,3]. This condition can be debilitating, affecting daily activities and overall quality of life [1,3]. The criteria for diagnosing PPPD, as established by the Bárány Society and the International Classification of Vestibular Disorders, include a history of persistent non-vertiginous dizziness lasting three months or more, exacerbated by upright posture or movement and accompanied by a heightened visual dependency or postural instability [3]. Patients may also exhibit symptoms such as generalized anxiety, avoidance of situations that provoke dizziness, and difficulty concentrating [3]. The diagnosis of PPPD is clinical, based on thorough history-taking and examination to exclude other vestibular or medical conditions [3,7]. Understanding these criteria is essential for healthcare professionals to accurately diagnose and manage PPPD, ultimately improving patient outcomes and quality of life [3,7].

In the late 19th century, German physicians described syndromes of dizziness and discomfort in motion-rich environments, associated with autonomic arousal, anxiety, and avoidance behaviors [3]. These included Platzschwindel (vertigo in a plaza or square), focusing on neuro-ophthalmologic processes, and Platzangst (fear in a plaza or square), emphasizing psychological origins [3]. Another syndrome, Die Agoraphobie (fear of the marketplace), linked postural control, locomotion, and spatial orientation [3]. These syndromes were debated as neurologic or psychiatric, but eventually faded from use, and agoraphobia became a psychiatric disorder without its original space and motion context [3]. Around the same time, space-motion discomfort (SMD) was described as a condition marked by uneasiness about spatial orientation and heightened sensitivity to motion stimuli, particularly in visually rich environments [3]. Visual vertigo (VV) was identified as a phenomenon in patients recovering from vestibular losses, manifesting as unsteadiness or dizziness triggered by complex visual stimuli, later termed visually induced dizziness (VID) [3]. Additionally, chronic subjective dizziness (CSD) was defined as persistent non-vertiginous dizziness or unsteadiness, increased sensitivity to motion, and difficulty maintaining visual focus [3]. These syndromes provided insights into the complex interactions between vestibular function, visual stimuli, and psychological factors in the experience of dizziness [3]. 

The Bárány Society criteria for PPPD are listed in Table 1. All five criteria must be fulfilled to make the diagnosis [3]. 

**Table 1:** Bárány Society criteria for PPPD PPPD: persistent postural-perceptual dizziness

One or more symptoms of dizziness, unsteadiness, or non-spinning vertigo are present on most days for three months or more. (1) Symptoms last for prolonged (hours-long) periods of time, but may wax and wane in severity. (2) Symptoms need not be present continuously throughout the entire day.
Persistent symptoms occur without specific provocation, but are exacerbated by three factors: (1) upright posture, (2) active or passive motion without regard to direction or position, and (3) exposure to moving visual stimuli or complex visual patterns.
The disorder is precipitated by conditions that cause vertigo, unsteadiness, dizziness, or problems with balance including acute, episodic, or chronic vestibular syndromes, other neurologic or medical illnesses, or psychological distress. (1) When the precipitant is an acute or episodic condition, symptoms settle into the pattern of criterion A as the precipitant resolves, but they may occur intermittently at first and then consolidate into a persistent course. (2) When the precipitant is a chronic syndrome, symptoms may develop slowly at first and worsen gradually.
Symptoms cause significant distress or functional impairment.
Symptoms are not better accounted for by another disease or disorder.

Pathophysiology and clinical features

The pathophysiology of PPPD is not fully understood, but it is believed to involve complex interactions between the sensory systems and central nervous system [1,4]. It encompasses dysfunction in the integration of visual, vestibular (related to balance and spatial orientation), and proprioceptive (awareness of body position) information [1,4].

Sensory Mismatch

The concept of sensory mismatch is a crucial aspect in understanding the pathophysiology of PPPD because it is often associated with the information received from these sensory systems [4]. This condition arises when there is a conflict between visual, vestibular, and proprioceptive inputs, leading to persistent dizziness and unsteadiness [4,7]. The brain struggles to integrate these conflicting signals, which can result in a constant feeling of motion or imbalance, even in the absence of actual movement [4,5,7]. Conflicting information between visual input and signals from the vestibular system can occur, especially during head movements or changes in posture [5]. Patients often experience increased symptoms in environments with complex visual stimuli, such as crowded places or while using screens [4,5]. This sensory overload can exacerbate the condition, making everyday activities challenging [4,5]. Normally, the brain can adapt to short-term sensory conflicts, and the mismatch is resolved through central processing and integration [4,5]. In PPPD, there may be a breakdown in this adaptive process, leading to a chronic and maladaptive response to sensory discrepancies [8].

Central Sensitization

It is a process in which the central nervous system becomes hypersensitive to stimuli, resulting in an exaggerated response to sensory input [5,9]. This phenomenon is associated with various chronic pain and neurological conditions, and it plays a significant role in the pathophysiology of PPPD [9]. Emotional factors, such as anxiety or stress, can contribute to this central sensitization [9]. In PPPD, the interplay between central sensitization and psychological factors can create a cycle where heightened emotional responses contribute to increased symptom severity and vice versa [9].

In a study comparing PPPD patients and healthy controls, vestibular stimulation was administered using a motorized rotary chair [10]. The findings indicated that PPPD patients have a reduced vestibulo-perceptual threshold, leading to increased motion sensitivity and a concomitant increase in vegetative responses, both of which are related to the duration of the disease [10]. This threshold may be influenced by psychological factors, such as anxiety [10]. Thus, the vestibular system appears to be susceptible to modulation, which is attributed to cortical-subcortical hyperexcitability, reduced sensory feedback, and a deficit in habituation, reflecting chronic maladaptation [10]. A decreased motion perception threshold, combined with abnormal vestibular responsiveness in PPPD patients, leads to motion intolerance and the induction of dizziness when exposed to movement [10].

Therapeutic approaches often include interventions aimed at desensitizing the central nervous system, reducing hypersensitivity, and promoting adaptive changes in neural processing [11]. Understanding the complex interplay between central sensitization and sensory processing is essential for developing effective treatment strategies for PPPD [12]. A comprehensive and multidisciplinary approach that addresses both the physical and psychological aspects of the condition is often recommended for managing PPPD [12].

Adaptation Mechanisms

Under normal circumstances, the brain is adept at adapting to changes in sensory input to maintain balance. For example, when a person moves their head, the visual and vestibular systems work together to provide a stable perception of the environment [13]. Maladaptive changes in how the brain processes sensory information may occur, leading to a persistent perception of dizziness even when there is no ongoing pathology [14]. The brain's attempts to adapt to perceived threats or imbalances might contribute to the chronic nature of PPPD [14].

Neurotransmitter Involvement

The role of neurotransmitters is an area of ongoing research, and while the exact mechanisms are not fully understood, there is evidence suggesting that neurotransmitter dysfunction may contribute to the development and persistence of symptoms in PPPD, for example, those involved in mood regulation and sensory processing may contribute to the development or perpetuation of symptoms [14].

It is crucial to acknowledge that research on PPPD is continuously evolving and the precise mechanisms involved are likely to become clearer with the advancement of scientific understanding [1,4]. The complex nature of PPPD, which involves both physiological and psychological components, underscores the necessity for a comprehensive approach in its diagnosis and management [1,4].

Individuals with PPPD experience a continuous and chronic sensation of dizziness or unsteadiness, often occurring daily [2,4]. This sensation is not solely triggered by specific head movements; it could result from an acute vestibular disorder, a stressful life event, or a neurologic injury and could also be worsened by an upright posture, passive movement, and exposure to complex visual stimuli [2,14]. PPPD may be precipitated by vestibular disorders, medical illnesses, or psychological distress that cause vertigo, dizziness, or unsteadiness [3].

Also, this condition is frequently associated with psychological factors such as anxiety and depression [15]. Emotional responses can influence the severity and persistence of symptoms, and these conditions often coexist [15]. This association has a significant impact on an individual's quality of life, leading to functional impairment, reduced daily activities, and increased healthcare utilization [16]. These key factors support that the treatment of PPPD often requires a holistic approach, addressing both physiological and psychological aspects [16], which is crucial for healthcare professionals to navigate the complexities and develop effective strategies for diagnosis and management [8,14].

Controversies

Major controversies surrounding PPPD primarily revolve around the duration of symptoms, overlap with other conditions, and treatment approaches.

Duration of Symptoms

Defining the duration necessary for a diagnosis of PPPD remains a point of contention [1-3]. Some researchers and clinicians might have differing opinions on the appropriate time frame for categorizing symptoms as persistent. They argue for a specific time frame, while others emphasize the importance of recognizing the chronic nature of the condition, irrespective of a predefined duration [1-3].

Overlap with Other Conditions

There is an ongoing discussion about the potential overlap between PPPD and other vestibular disorders, migraines, and psychological conditions [17]. Distinguishing PPPD from these related conditions poses a challenge in both research and clinical settings [17]. Studies have provided evidence of an association between PPPD and migraine headaches [18]. Sarna et al. demonstrated that most patients with PPPD meet many of the criteria for migraine headaches [18]. They found a 53% prevalence of migraine headaches among individuals with PPPD, compared to the 8-13% prevalence of migraine headaches in the general population [18]. This significant difference suggests a strong association between PPPD and migraine headaches [18]. Migraine and PPPD share several characteristics, such as being attributed to cortical disturbances, manifesting hypersensitivity to various sensory stimuli (including visual motion, motion, light, sounds, and smells), and exhibiting a female predominance [18]. Therefore, identifying the coexistence of both disorders is crucial, as treating the migraine may reduce the severity of symptoms in PPPD [18].

Anxiety disorders, such as generalized anxiety disorder and panic disorder, often coexist with PPPD [19]. There are ongoing discussions regarding whether these factors are causative or secondary to persistent dizziness [19]. The relationship is bidirectional, with anxiety potentially worsening dizziness symptoms and chronic dizziness contributing to heightened anxiety [20]. Anxiety disorders can manifest with persistent postural dizziness in various ways [20]. One manifestation involves triggering episodes of dizziness and unsteadiness crises [20]. This is due to the heightened emotional state associated with these disorders, which amplifies the symptoms [20]. Additionally, living with persistent dizziness and a sense of imbalance can be distressing [20]. It may lead individuals to develop anxiety as a reaction to their ongoing symptoms and the impact these symptoms have on their daily lives [20].

Also, both PPPD and anxiety can involve the dysregulation of the autonomic nervous system and other neurological processes [4]. Shared neurobiological factors may contribute to the co-occurrence of these conditions [4].

Treatment Approaches

There isn't necessarily controversy surrounding the treatment of PPPD; there may be ongoing discussions and debates about the most effective strategies [21,22]. Since vestibular rehabilitation, which involves exercises to improve balance and reduce dizziness, is a main component of PPPD treatment, controversies may involve the optimal duration, intensity, and specific exercises used in vestibular rehabilitation for PPPD [21,22]. Pharmacotherapy agents, such as selective serotonin reuptake inhibitors (SSRIs) or vestibular suppressants, may be considered in the management of PPPD [23]; some dissension involves the potential benefits, risks, and appropriate duration of medication use [23]. Addressing these controversies is crucial for refining diagnostic criteria, improving treatment outcomes, and advancing our understanding of PPPD [23]. Ongoing research aims to unravel these complexities, providing a clearer framework for clinicians and researchers working with individuals affected by this challenging condition [21-23].

Challenges

Challenges with Medications

Medications, such as vestibular suppressants or anxiolytics, may provide only partial relief, and their efficacy varies among individuals with PPPD [21-23]. Achieving consistent and significant improvement can be challenging. Also, many medications prescribed for PPPD come with potential side effects, ranging from drowsiness to cognitive impairment [23]. Balancing the benefits and drawbacks of medications poses a challenge, particularly when side effects impact daily functioning [23].

Individual responses to medications exhibit a high degree of variability, making it imperative to recognize that the efficacy of a particular treatment for one individual may not translate to effectiveness for another [24]. The identification of the optimal medication and dosage often necessitates a meticulous trial-and-error approach [24]. This method, while essential for achieving therapeutic success, can contribute to the lengthening of the overall treatment duration [24].

Pharmacotherapeutic strategies targeting anxiety or depression, frequently concomitant with PPPD, are often successful in addressing psychological manifestations but may not directly ameliorate the underlying vestibular-related issues [1,23]. The synergistic implementation of psychological and pharmacological interventions presents intricate challenges in achieving optimal therapeutic outcomes.

Challenges with Vestibular Rehabilitation

Vestibular rehabilitation emerges as a pivotal component in the multifaceted management of PPPD [25]. This therapeutic modality assumes a central role in addressing the intricate interplay of vestibular dysfunctions contributing to the chronic dizziness characteristic of PPPD [25]. The efficacy of vestibular rehabilitation hinges on unwavering commitment and disciplined, consistent practice [26,27]. Adherence to prescribed exercises poses a notable challenge, particularly during the initial phases when individuals may contend with discomfort or perceive a gradual pace of improvement [26]. Clinicians navigating the landscape of PPPD therapeutics must underscore the imperative of sustained engagement in vestibular rehabilitation to optimize its impact on vestibular function and mitigate the enduring symptomatology associated with this complex condition [27].

Responses to vestibular rehabilitation vary widely among individuals. Some may experience significant improvement, while others may find only modest relief [27]. Determining the most effective exercises for each person remains a complex aspect of treatment [27]. Individuals with PPPD may have heightened sensitivity to certain movements, making it challenging to engage in exercises that induce dizziness or discomfort. Tailoring rehabilitation programs to accommodate individual tolerances is crucial but can be intricate [27].

**Figure 1:**
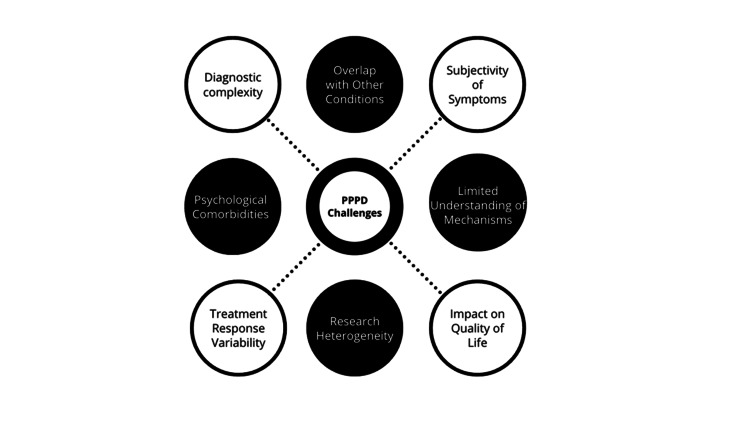
Challenges in PPPD PPPD: persistent postural-perceptual dizziness Image Credit: Authors

PPPD often involves both physiological and psychological components. Coordinating vestibular rehabilitation with other therapeutic modalities, such as cognitive-behavioral therapy (CBT), poses challenges in ensuring a comprehensive and integrated approach [27,28]. In cases where PPPD coexists with other vestibular disorders or medical conditions, tailoring vestibular rehabilitation becomes more complex [27,28]. Addressing multiple contributing factors requires a nuanced and individualized treatment plan [27,28]. Navigating these challenges underscores the importance of a personalized and multidisciplinary approach to PPPD treatment, where healthcare professionals collaborate to address the diverse aspects of this complex condition. Integrating medications, CBT, and vestibular rehabilitation, with a focus on individual needs and tolerances, is crucial for optimizing outcomes [27,28]. A summary of PPPD challenges is seen in Figure 1.

Challenges with CBT

CBT faces unique challenges when applied to patients with PPPD, primarily due to the intricate interplay between the physical symptoms and psychological aspects of the disorder [29]. While CBT is recognized for its effectiveness in managing anxiety and depression, which are commonly associated with PPPD, translating these benefits to the physical symptoms of dizziness and imbalance can be complex [29]. Patients may experience difficulty in engaging with CBT due to the pervasive nature of their physical symptoms, which can affect concentration and the ability to participate in therapy sessions [25,29]. Additionally, the somatic focus of PPPD symptoms might require therapists to adapt traditional CBT techniques to address the unique needs of this population, integrating specific strategies aimed at reducing symptom-related anxiety and improving coping mechanisms [29]. The lack of widespread understanding and recognition of PPPD in the broader healthcare community can also limit access to appropriately trained therapists, further complicating the treatment landscape [29]. Thus, while CBT holds potential as a component of a comprehensive treatment plan for PPPD, overcoming these challenges is essential to maximize its efficacy and improve patient outcomes [29].

PPPD and functional impairment

Functional Impairment

The chronic nature of PPPD can have a profound impact on an individual's daily life, restricting their capacity to engage in routine activities [16]. Functional impairment may extend to various aspects, affecting work, social interactions, and recreational pursuits. Individuals with PPPD commonly endure persistent, non-vertiginous dizziness exacerbated by upright posture and head movements [6]. This ongoing sensation of unsteadiness, imbalance, and fear of falling can significantly hinder a person's ability to carry out daily activities that demand balance and coordination [30,31]. The consequences of these symptoms highlight the substantial challenges faced by individuals living with PPPD in their day-to-day lives [30,31].

Reduced Quality of Life

The combined impact of the mentioned factors significantly diminishes the overall quality of life for individuals dealing with PPPD [30]. Beyond affecting specific activities, functional impairment extends into various aspects of daily life [30]. The persistent symptoms of PPPD cast a pervasive influence, leaving an impact on emotional well-being, imposing physical limitations, and intricately influencing social relationships [30]. Managing chronic, non-vertiginous dizziness intensified by upright posture and head movements can create a sustained sense of unease, affecting mental health [1,30,31]. Additionally, the intricate interplay of PPPD symptoms with social relationships compounds the challenges, potentially fostering a sense of isolation and hindering the ability to fully participate in interpersonal connections [1,30,31]. Collectively, these diverse aspects contribute to a nuanced and comprehensive understanding of the reduced overall sense of well-being experienced by individuals with PPPD in daily life [6,30]. Understanding the multifaceted impact of PPPD on quality of life is essential for healthcare professionals to develop comprehensive treatment plans that address not only the physical symptoms but also the emotional and social aspects of the condition [6,30]. Holistic care that considers the individual's overall well-being is key to improving their quality of life [6,30].

Treatment Response Variability

Responses to treatment, whether pharmacological, rehabilitative, or psychological, can vary widely among individuals. Predicting and ensuring a positive response to interventions remains a challenge in managing PPPD effectively [31]. Recognizing these challenges is crucial for healthcare professionals to develop tailored approaches that address the diverse clinical manifestations and limitations associated with PPPD [31]. A comprehensive understanding of the disorder's impact is essential for providing patient-centered care and improving outcomes [31].

Balancing Work, Health, and Finances

Those affected by PPPD face a broad question: to find the stable work performance paralleled by potential complications of concentration and a necessity to make many breaks [14]. PPPD and its accompanying struggle will on most occasions significantly increase the utilization of healthcare services as this is done to diagnose as well as curb the pains, necessitating increased consultations and interventions [1,6]. As a result, the overall combination of medical costs, possible risks at work, and the necessity for alternative therapies results in much economic strain not only on an individual but also on his or her family members [30].

Proposals for the future

Integrated Research Approaches

Promoting interdisciplinary research that integrates neurology, otolaryngology, psychiatry, and rehabilitation sciences is imperative for advancing our understanding of PPPD [32]. The collaboration of experts from these diverse disciplines contributes nuanced perspectives to elucidate the multifaceted nature of PPPD comprehensively [32]. An interdisciplinary approach facilitates a holistic exploration of PPPD, considering not only its physiological underpinnings but also its psychological and rehabilitative aspects [32]. This collaborative synergy of expertise is crucial for deciphering the complexities of PPPD and holds the potential to generate innovative, patient-centered interventions [32]. Encouraging interdisciplinary research is instrumental in fostering transformative breakthroughs that not only enhance our understanding of PPPD but also elevate the standard of care provided to individuals contending with this challenging condition [32].

Longitudinal Studies

Advocating for ongoing, in-depth studies that follow individuals with this condition over the long term is crucial for truly understanding how this condition unfolds and evolves [33]. Longitudinal studies, characterized by their extended temporal scope, afford a distinctive opportunity to capture the dynamic temporal features of PPPD, elucidating its manifestation patterns, fluctuations, and potential resolutions or transformations [33]. Through methodical data collection over an extended temporal horizon, researchers can discern nuanced patterns, identify triggering factors, and delineate diverse trajectories of PPPD [33]. Committing resources to longitudinal research not only contributes to the scientific elucidation of PPPD but also pledges advancements in patient care by offering evidence-based insights that can guide bespoke interventions and management [33].

Treatment Optimization Trials

Conducting randomized controlled trials to optimize and compare various treatment modalities, including medications, vestibular rehabilitation, and psychological interventions, can help establish evidence-based guidelines for effective PPPD management [23].

Telemedicine Solutions, Educational Initiatives, and Patient Support Networks

Exploring and advancing telemedicine solutions for PPPD facilitates remote monitoring, consultations, and follow-ups, thereby addressing challenges related to geographical and mobility constraints [34]. This initiative not only enhances patient access to specialized care but also fosters a more patient-centric and inclusive healthcare environment [33]. Concurrently, launching educational campaigns is pivotal in raising awareness among healthcare professionals, patients, and the general public, leading to early recognition, reduced stigma, and increased support for individuals grappling with PPPD [34]. Moreover, fostering international collaboration in PPPD research is imperative, as it facilitates the exchange of diverse perspectives, methodologies, and findings, ultimately accelerating progress in comprehending and managing this intricate disorder on a global scale [34]. The establishment and promotion of support networks for individuals with PPPD, including online communities and educational resources, contribute significantly to coping strategies and shared experiences, fostering a sense of community among those affected [34]. This integrated approach reflects a commitment to advancing PPPD care through innovation, education, collaboration, and support [34].

Trends and findings

Increased Recognition and Research Focus

In recent years, there has been a notable increase in the recognition and research focus on PPPD as a distinct clinical entity [17]. This surge in attention reflects a growing awareness of the significant impact PPPD has on patients' lives and the need for more targeted treatments [17]. Researchers have directed their efforts toward understanding various aspects of PPPD, including its prevalence, clinical characteristics, and underlying mechanisms [3,17].

Studies investigating the prevalence of PPPD have revealed its relatively high occurrence, highlighting the importance of addressing this condition in clinical practice [17]. Additionally, research has sought to identify the clinical features that distinguish PPPD from other vestibular and psychological disorders, aiding in more accurate diagnosis and treatment planning [3,17]. Moreover, efforts to uncover the underlying mechanisms of PPPD have shed light on the complex interplay between sensory inputs, central processing, and psychological factors [3,17]. This deeper understanding is crucial for developing effective therapeutic strategies that target the root causes of PPPD [3,17].

Neuroimaging Studies

Advances in neuroimaging techniques have revolutionized researchers to explore structural and functional brain changes associated with PPPD [32]. Functional magnetic resonance imaging (fMRI) has been instrumental in uncovering structural changes in the brains of individuals with PPPD [33]. Researchers can identify alterations in specific regions, such as the vestibular system, cerebellum, and cortical areas, which play crucial roles in spatial orientation and postural control [34]. The findings from these studies suggest that brain regions in individuals with PPPD may not be as active or as well-connected as in healthy individuals [33]. This diminished activity and connectivity could lead to poorly integrated mechanisms for posture and gaze control [33,35].

Investigators using fMRI in patients with PPPD have observed decreased functional connectivity in the temporal lobe but enhanced connectivity in the occipital lobe [35]. These findings indicate that the interaction between the visual cortex and the vestibular cortex is abnormal, with this interaction being predominantly driven by visual information [36]. This abnormality explains the impaired ability of individuals with PPPD to adjust posture and movement using both vestibular and visual information [35,36]. It also accounts for their visual dependence and the dizziness experienced following exposure to complex visual stimuli [33,35]. Also, there have been described some changes in brain function registered by magnetoencephalography (MEG) in a study where MEG recordings from patients with PPPD and control subjects were analyzed [36]. The frequency-dependent alterations in neuromagnetic activity were observed in patients with PPPD compared with healthy controls [36].

PPPD represents a challenging frontier in the field of vestibular medicine, characterized by its multifaceted symptomatology, complex pathophysiology, and significant impact on patients' quality of life [1]. Despite advancements in understanding its clinical presentation and associations with other conditions such as migraines and anxiety disorders, PPPD remains an enigmatic condition [3,5]. Emerging research, particularly in neuroimaging, offers promising insights into PPPD's neurobiological basis, potentially paving the way for more targeted therapies [34]. However, the variability in treatment responses and the ongoing challenges over treatment and rehabilitation highlight the need for continued investigation and collaboration across specialties [8]. Future research should focus on longitudinal studies to understand PPPD's progression and on optimizing treatment strategies through randomized controlled trials. The management of PPPD, therefore, necessitates a patient-centered approach, recognizing the diversity of symptoms and their impact on individuals' lives.

## Conclusions

PPPD is a complex condition that blurs the lines between physiological and psychological health, presenting significant challenges in diagnosis and treatment. The current body of research underscores the necessity of a multidisciplinary approach to manage PPPD effectively, integrating pharmacotherapy, vestibular rehabilitation, and psychological support tailored to individual patient needs.
